# Ecological Succession of Sulfur-Oxidizing *Epsilon*- and *Gammaproteobacteria* During Colonization of a Shallow-Water Gas Vent

**DOI:** 10.3389/fmicb.2018.02970

**Published:** 2018-12-06

**Authors:** Sushmita Patwardhan, Dionysis I. Foustoukos, Donato Giovannelli, Mustafa Yücel, Costantino Vetriani

**Affiliations:** ^1^Department of Marine and Coastal Sciences, Rutgers University, New Brunswick, NJ, United States; ^2^Geophysical Laboratory, Carnegie Institution of Washington, Washington, DC, United States; ^3^Institute of Marine Science, National Research Council, Ancona, Italy; ^4^Earth-Life Science Institute, Tokyo Institute of Technology, Tokyo, Japan; ^5^Institute of Marine Sciences, Middle East Technical University, Mersin, Turkey; ^6^Department of Biochemistry and Microbiology, Rutgers University, New Brunswick, NJ, United States

**Keywords:** shallow-water gas vent, Tyrrhenian Sea, Tor Caldara, microbial biofilms, active microbial communities, *Epsilonproteobacteria*, geothermal

## Abstract

In this study, we integrated geochemical measurements, microbial diversity surveys and physiological characterization of laboratory strains to investigate substrate-attached filamentous microbial biofilms at Tor Caldara, a shallow-water gas vent in the Tyrrhenian Sea. At this site, the venting gases are mainly composed of CO_2_ and H_2_S and the temperature at the emissions is the same as that of the surrounding water. To investigate the composition of the total and active fraction of the Tor Caldara biofilm communities, we collected established and newly formed filaments and we sequenced the 16S rRNA genes (DNA) and the 16S rRNA transcripts (cDNA). Chemoautotrophic sulfur-oxidizing members of the *Gammaproteobacteria* (predominantly *Thiotrichales*) dominate the active fraction of the established microbial filaments, while *Epsilonproteobacteria* (predominantly *Sulfurovum* spp.) are more prevalent in the young filaments. This indicates a succession of the two communities, possibly in response to age, sulfide and oxygen concentrations. Growth experiments with representative laboratory strains in sulfide gradient medium revealed that *Sulfurovum riftiae* (*Epsilonproteobacteria*) grew closer to the sulfide source than *Thiomicrospira* sp. (*Gammaproteobacteria, Thiotrichales*). Overall, our findings show that sulfur-oxidizing *Epsilonproteobacteria* are the dominant pioneer colonizers of the Tor Caldara biofilm communities and that *Gammaproteobacteria* become prevalent once the community is established. This succession pattern appears to be driven - among other factors - by the adaptation of *Epsilon*- and *Gammaproteobacteria* to different sulfide concentrations.

## Introduction

Hydrothermal and gas vents are manifestations of volcanism on earth. Both environments are characterized by venting of gas and/or hydrothermal fluids, enriched in reduced, inorganic chemical species, into the oxygenated water column. This creates a redox disequilibrium, which is then harnessed by the prokaryotic communities to convert chemical energy into ATP. Both types of vents have been known to occur at varying depths, from intertidal regions to abyssal depths (Tarasov et al., 2005). Vents that occur at depths ≤ 200 meters were traditionally classified as “shallow-water” hydrothermal vents, and the ones that occur at depths greater than 200 meters were classified as “deep-sea” hydrothermal vents (Tarasov et al., 2005). Shallow-water vents (both hydrothermal and gas) have been discovered at seamounts, submarine volcanoes, volcanic arcs and back arcs (Price and Giovannelli, 2017). Common features of both shallow-water and deep-sea vent ecosystems include high concentrations of reduced compounds, gases (e.g., H_2_, CH_4_, H_2_S) and heavy metals. However, the temperature of hydrothermal fluids at shallow-water vents isn’t as high as that of the deep-sea vents, where it can exceed 400°C (Fornari et al., 1998). Shallow vents are also affected by input of meteoric water and terrigenous organic carbon. At most shallow-water vents, it has been observed that H_2_ content of the venting fluid-gas is exceedingly low as compared to deeper vents (Tarasov et al., 2005). Along with geochemical energy, the availability of light energy leads to co-occurrence of photoautotrophy and chemoautotrophy, setting these shallow systems apart from their deep-sea counterparts. In the broader context, coastal geothermal environments can serve as model systems to study the effects of ocean acidification and increasing temperatures in the oceans. Further, high levels of potentially toxic heavy metals have been associated with shallow-water vents, making them probable sources of coastal pollution leading to bioaccumulation and biotransformation of these metals in the organisms living in the vicinity (Price and Giovannelli, 2017).

The collision of the African and European tectonic plates leads to the subduction of the oceanic part of the African plate below Europe, which causes hydrothermalism in the Mediterranean Sea (Dando et al., 1999). The resulting volcanic arcs and back arcs are found in the Aegean and Tyrrhenian Seas (e.g., the Aeolian Islands arc). Hydrothermal venting in volcanic arc systems is characterized by the release of volcanic gases. Volcanic arc venting is a combination of degassing from subducted slab and mantle as well as decomposition of the carbonaceous sediment on top (Dando et al., 1999). While the terrestrial volcanic systems of the Mediterranean region have been well studied because of their impact on local population, the submarine component started receiving attention only in the 1990s (Dando et al., 1999). The Tyrrhenian margin of Southern and Central Italy is a volcanic geothermal region with a thinned continental crust. It is characterized by heat flows and degassing of CO_2_ of deep magmatic/mantle origin due to the alkali-potassic volcanoes present in the region from the Quaternary period (Carapezza et al., 2012). One of the relatively recent volcanoes is the Colli Albani, whose eruptive activity began 600 ka years ago and went on till about 5.8 ka (Holocene). Although the age of its most recent eruption is controversial, there is unanimity about its quiescent status (Carapezza et al., 2012; Marra et al., 2016). Due to the infiltration of cold meteoric water, there aren’t any thermal springs or fumaroles in the immediate vicinity of the Colli Albani volcano. However, there are several gas emission sites associated with the flanks of this volcanic system near the coast of the Tyrrhenian sea, Tor Caldara being one of them (Carapezza et al., 2012). The helium isotopic composition of the gases released from Colli Albani suggests a likely origin from a deep magma source affected by crustal contamination during its primary generation in a subduction process (Carapezza and Tarchini, 2007).

Till date, the microbiology of shallow-water hydrothermal vents has been investigated at the Aeolian Islands and Milos in the Mediterranean Sea, at the Azores and Iceland in the Atlantic Ocean, and at White Point, New Caledonia, Papua New Guinea, Kueishan and Taketomi islands in the Pacific Ocean (Sievert et al., 1999, 2000a,b; Marteinsson et al., 2001; Kalanetra et al., 2004; Hirayama et al., 2007; Giovannelli et al., 2013; Meyer-Dombard et al., 2013; Quéméneur et al., 2014; Gugliandolo et al., 2015; Wang et al., 2017). Previous shallow-water vent microbial diversity studies in the Tyrrhenian Sea have mostly been focused on vents off the coast of the Aeolian Islands. However, none of these studies investigated the active fraction of the microbial communities and very few carried out physiological studies of bacteria isolated from these vents. These previous studies have revealed that, within the *Proteobacteria*, thermophilic as well as mesophilic chemoautotrophic members belonging to the *Gammaproteobacteria* and *Epsilonproteobacteria* dominate these sites, whereas members of the *Deltaproteobacteria* class are abundant in the sediments. Occurrence of phototrophic members belonging to *Alphaproteobacteria*, *Cyanobacteria*, *Chlorobi* and *Chlorofexi* were recorded in addition to thermophilic heterotrophs belonging to the *Firmicutes* phylum and members of the *Euryarchaeota* and *Crenarchaeota* (Gugliandolo and Maugeri, 1993, 1998; Gugliandolo et al., 2006, 2015; Maugeri et al., 2008, 2010, 2013; Lentini et al., 2014).

In the present study, we used next generation 16S rRNA amplicon sequencing, geochemical analyses and physiological studies of pure cultures to investigate the abundance, diversity and adaptation to sulfide of the filamentous microbial biofilms as well as the gas composition of the shallow-water gas vents at Tor Caldara.

## Materials and Methods

### Study Site

Tor Caldara is a natural reserve located near the town of Anzio, around 60 km south of Rome and 40 km south-west of the Colli Albani volcano. It sits on a buried carbonate basement that has been hypothesized as one of the sources of CO_2_ at this site (Carapezza et al., 2012). The study site (41°29′ 9″ N 12°35′ 23″ E) is a submarine gas vent located at a water depth of approximately 3 m off the coast of the natural reserve. Along the coastline, hydrothermal alterations of the rocks and soil are visible in correspondence with the study site. A significant amount of degassing can be observed underwater and at the surface. The rocks occurring on the sandy seabed in the vicinity of the gas discharges are colonized by filamentous microbial communities (Supplementary Figure 1a).

### Collection of Biological Samples

Samples were collected by a SCUBA diver in the summer (month of August) over a course of 5 years from 2012 to 2016. Sampling was unsuccessful in 2014 due to inclement weather conditions. Samples of established filaments (EF from here on) growing on rocks in the vicinity of the gas emissions were collected in all 4 years (2012, 2013, 2015, and 2016). A total of four samples (one per each year) were used for downstream applications. In 2012, filament samples were collected by scraping them off the rocky substrate; in the following years (2013, 2015, and 2016) filament samples were collected using a 60 ml sterile syringe. Briefly, the syringe plunger was retracted to generate vacuum and draw the filaments into the barrel. Once the sampling was completed, the syringe was inverted to let the filament deposit onto the plunger seal and the excess seawater was slowly pushed out of the barrel. The remaining filaments were pushed into a sterile tube containing RNA Later. To investigate the early stage of substrate colonization, in 2016 we performed a biofilm colonization experiment by mounting several sterile glass slides very close to each other on an aluminum rod at the venting site for 4 days (Supplementary Figure 1b). The young filaments (YF from here on) growing on these slides were then collected for further analyses. All samples were stored in ambient seawater at 4°C to be used as inocula for cultivation work and in RNA Later at −80°C for further nucleic acid extraction. The EF and YF were also stored in 2% formaldehyde at 4°C for microscopy.

### Collection of Gas Samples

In 2015, we built an in-house apparatus for collecting gas samples (10 replicates) at Tor Caldara. A weighted inverted funnel was deployed over the gas discharges. The gases trapped inside the inverted funnel were conveyed via connected tubing to a gas-tight valve, which was finally connected to a syringe. Twenty milliliters of gas were collected and injected into 25 ml stoppered borosilicate vials containing 4 ml of 6% (w/v) AgNO_3_, 25% (w/v) H_3_PO_4_ and 20 ml Argon grade zero. The vials were pretreated by following protocols discussed elsewhere (Foustoukos, 2012). The precipitated Ag_2_S and gases in the headspace was measured and analyzed. Concentrations of H_2(g)_, CO_(g)_, CO_2(g)_, and CH_4(g)_ were measured by a SRI 8610C gas chromatograph equipped with TCD/FID detector and a Carboxen-1010 Plot/Silica-Gel column. The detection limit for these volatiles is 1–5 μmol/kg (<0.001 mol%) (Foustoukos et al., 2011). Analytical errors are within 1–15% (2σ) and reflect the larger values of errors estimated either by instrument calibration and/or by duplicate analysis of individual samples. The precipitated Ag_2_S in the solution was filtered on to a 0.2 μm polycarbonate and weighed. Temperature and pH (using an Oakton waterproof pH/temperature probe) and salinity (using a Spartan A 366 ATC refractometer) measurements were also taken in the venting area as well as 100 m away from venting in 2015.

### Microscopy and Elemental Composition of the Filament Communities

Biofilm samples, as well as manually teased individual filaments stored in 2% formaldehyde, were mounted on aluminum pin stubs using double sided carbon adhesives for microscopy. Scanning electron micrographs were obtained for EF and YF using a Phenom ProX table top scanning electron microsope (SEM) at a voltage of 10 kV. Spot analysis using the Elemental Mapping & Line Scan software was performed at 15 kV using the same instrument to examine the elemental composition of the sample.

### Nucleic Acid Extraction

Both DNA and RNA were extracted from samples stored in RNA Later following a phenol:chloroform extraction protocol to study the total and active communities, respectively. Samples were centrifuged at 10,000 rmp for 3 min to remove the RNA Later supernatant. 0.8 g (wet weight) of the pelleted filaments was added to 850 μl extraction buffer (50 mM Tris-HCl, 20 mM EDTA, 100 mM NaCl; pH 8.0) supplemented with 100 μl of lysozyme (100 mg/ml) and incubated at 37°C for 30 min. This mix was then supplemented with 5 μl of proteinase K (20 mg/ml) and incubated at 37°C for 30 min followed by the addition of 50 μl SDS (20%) and further incubated at 65°C for 1 h. Nucleic acids were extracted by performing a series of phenol:chloroform: isoamylalcohol (25:24:1) and chloroform:isoamyl alcohol (24:1) extractions. Phenol:chloroform:isoamylalcohol (25:24:1) at pH 4.3 was used for RNA extractions. Multiple replicates of the same sample were co-extracted to reduce potential bias. The final supernatant was precipitated in 3 M sodium-acetate and isopropanol, washed twice with 70% ice cold ethanol and resuspended in ultra-pure water. For RNA preparation, carryover DNA was removed by treating the RNA with the TURBO DNase kit (Invitrogen, Carlsbad, CA, United States), according to the manufacturer’s specifications. This DNase treated RNA was reverse transcribed into cDNA using the Invitrogen cDNA synthesis kit (Invitrogen, Carlsbad, CA, United States), according to the manufacturer’s specifications. The quality of the genomic DNA and cDNA was assessed by polymerase chain reaction amplification of the 16S rRNA gene using primers Bact 8F (5′-AGAGTTTGATCCTGGCTCAG-3′) and Univ 519R (5′-ATTACCGCGGCTGCTGG-3′). PCR positive (DNA template known to produce an amplicon) and negative (no DNA template) controls were included, but not sequenced.

### 16S rRNA Gene Amplification and Sequencing

The DNA and cDNA from the Tor Caldara microbial communities were used as templates to amplify the variable 4 (V4) region of the 16S rRNA gene and transcript, respectively, using the prokaryotic universal primers (515F 5′-GTG CCA GCM GCC GCG GTA A-3′ and 806R 5′-GGA CTA CVS GGG TAT CTA AT-3′ (Caporaso et al., 2011). Amplicons were sequenced using the PGM Ion Torrent platform at the Molecular Research LP, Shallowater, TX, United States. Multiple PCR reactions for each sample were combined to reduce potential bias. Sequences are available through the NCBI Short Read Archive database with accession number SRP167116. Sequences were first depleted of barcodes and primers, then sequences < 150 bp, those with ambiguous base calls and with homopolymer runs exceeding 6 bp were removed. The amplicon size obtained was around 300 bp. The total number of reads for 16 samples after the chimera check was 1,311,749. The average number of reads per sample was 81,984.

### Bioinformatic Analyses

The 16S rRNA gene sequences were analyzed using QIIME 1.9 (Caporaso et al., 2010). Chimeric sequences were removed using ChimeraSlayer (Haas et al., 2011). Operational Taxonomic Units (OTUs) were picked at 97% similarity using the “pick_open_reference_otus.py” which clusters reads against the Greengenes v13_8 database (DeSantis et al., 2006). Reads that did not hit the database were subsequently clustered *de novo*. This method was chosen based on the assumption that the study site might harbor communities that are not well represented by the reference database. The OTUs were then taxonomically classified using the Ribosomal Database Project Classifier against the 2013 Greengenes database (Wang et al., 2007). OTUs that were not resolved up to the genus level were manually checked using NCBI BLAST (Altschul et al., 1990).

### Phylogenetic Analyses

Sequences of the V4 region of 16S rRNA genes of OTUs, full length 16S rRNA sequences of their closest cultured relatives (obtained by searching the NCBI non-redundant database by nucleotide BLAST search), and full length 16S rRNA sequences of cultured representatives from this study were aligned using Clustal Omega (Sievers et al., 2011) within SEAVIEW (Galtier et al., 1996; Gouy et al., 2010). The resulting alignment was used to reconstruct phylogenetic trees using the Maximum Likelihood algorithm with PhyML (Guindon and Gascuel, 2003) using the general time reversible (GTR) model, aLRT scores calculated.

### Statistical Analyses

The contribution of *Gammaproteobacteria* and *Epsilonproteobacteria* in each biofilm sample was further investigated using a *Gammaproteobacteria* to *Epsilonproteobacteria* ratio (GE ratio) calculated using the relative abundance of each class as follows: (%*Gammaproteobacteria* – %*Epsilonproteobacteria*)/ (%*Gammaproteobacteria* + % *Epsilonproteobacteria*). The obtained index is constrained between 1 (only *Gammaproteobacteria* present; *Epsilonproteobacteria* absent) and −1 (only *Epsilonproteobacteria* present), with a value of 0 representing equal relative amount of *Gamma*- and *Epsiloproteobacteria* (Supplementary Table 1). Since the majority of *Gamma*- and *Epsiloproteobacteria* were sulfur-oxidizing bacteria, this GE ratio was used to understand their shift in the YF and EF communities. The GE ratio was calculated for all the biofilm samples and differences among groups were tested by means of two-way analysis of variance (ANOVA) using the R statistical software (R: The R Project for Statistical Computing, 2018). Where ANOVA assumptions were rejected, a more conservative level of *p* was chosen. Both the biofilm age (established vs. young) and metabolic state (active vs. total communities) were used as factors in the analysis as well as the interaction term (age by metabolic state). In case of significant differences, a HSD Tukey *post hoc* test was performed.

### Isolation of Microorganisms

All primary enrichment cultures was set up by inoculating a slurry containing filament samples (stored at 4°C) re-suspended in 1 ml of artificial seawater into 10 ml of liquid medium or/and in 25 ml of solidified medium. A variety of different media were used, selecting for chemoautotrophic and heterotrophic microorganisms. Primary enrichments in liquid media were diluted to extinction and incubated at 30°C. Aliquots from the last dilution tube with growth were inoculated into fresh medium and pure cultures were obtained by performing three consecutive series of dilutions to extinction. During the isolation procedures, the cultures were incubated at 30°C.

#### Isolation of Heterotrophic Bacteria

Isolation of heterotrophs was targeted using full strength (FS) and low strength (LS) artificial seawater medium (ASW). FS ASW medium contained (per liter): 24 g NaCl, 0.7 g KCl, 7.0 g MgCl2, 3 g yeast extract, and 2.5 g peptone. LS ASW had the same amounts of salts per liter but lower amount of yeast extract (0.15 g) and peptone (0.125 g). For solid FS ASW and LS ASW, medium was supplemented with 15 g of agar per liter. Sulfide-oxidizing heterotrophs were isolated in sulfide gradient agar plugs (S-plugs) with yeast extract. S-plugs were prepared in 20 ml stoppered Hungate tubes (Bellco Glass). The S-plug medium contained a 3 ml solid (1.8% agar) sulfide reservoir (6.6 mM Na_2_S) on the bottom with 7 ml of solid (5% agar) THST medium (Podgorsek et al., 2004) with 0.05% yeast extract added on top.

#### Isolation of Chemolithoautotrophic Bacteria

Isolation of sulfur-oxidizing chemolithoautotrophs was carried out using liquid 1011 medium (Inagaki, 2004), which contained (l^−1^): 30 g NaCl, 0.14 g K_2_HPO_4_, 0.14 g CaCl_2_ ⋅ 2H_2_O, 3.4 g MgSO_4_ ⋅ 7H_2_O, 4.18 g MgCl_2_ ⋅ 6H_2_O, 0.33 g KCl, 0.5 mg NiCl_2_ ⋅ 6H_2_O, 0.5 mg Na_2_SeO_3_ ⋅ 5H_2_O, 0.01 g Fe(NH_4_)_2_(SO_4_)_2_ ⋅ 6H_2_O, 0.25 g NH_4_Cl, 1.5 g NaHCO_3_, 1.5 g Na_2_S_2_O_3_ ⋅ 5H_2_O, 10 ml trace mineral solution and 1 ml of trace vitamins. The medium was supplemented with a gas phase of CO_2_/O_2_ (95:5; 200kPa), and 10% (w/v) potassium nitrate under a N_2_/CO_2_ gas phase (80:20; 200 kPa), for isolating aerobic and anaerobic sulfur-oxidizing chemoautotrophs, respectively. Sulfide-oxidizing chemolithoautotrophs were isolated using S-plugs (as described in the previous paragraph) without yeast extract. The THST medium of the S-plugs was amended with 100 μl/10 ml of potassium nitrate (500 mM) and ammonium ferric citrate (500 mM) to enrich for anaerobic sulfide-oxidizers.

The isolation of *Thiothrix*-like bacteria was attempted (unsuccessfully) using *Thiothrix* growth medium (DSMZ medium 573), which contained (per liter): 0.20 g NH_4_Cl, 0.01 g K_2_HPO_4_, 0.01 g MgSO_4_, 20 ml CaSO_4_, 5 ml trace element solution from DSMZ medium 155, 0.10 g of Na-acetate, 12 g agar and 0.3 g Na_2_S.

### Growth of *Sulfurovum riftiae* and *Thiomicrospira* spp. in Sulfide Gradients and Generation of Sulfide Concentration Profiles

S-plugs for growth of *Thiomicrospira* sp. strain EPR 85 (Houghton et al., 2016) and *Sulfurovum riftiae* DSM 101780 (Giovannelli et al., 2016) were prepared as described above. However, the THST medium was replaced with medium 1011 without sodium thiosulfate (Inagaki, 2004). Replicate S-plug tubes were inoculated with cultures individually. The inoculated tubes along with un-inoculated controls were incubated at 30°C. *Thiomicrospira* sp. strain EPR 85 and *S. riftiae* were grown under a gas phase of CO_2_/O_2_ (95:5; 200 kPa) and N_2_/CO_2_ (80:20; 200 kPa), respectively. The upward diffusion of sulfide from the reservoir into the medium created a gradient of decreasing sulfide concentrations, thus providing micro-niches for selective growth of the two strains. A voltammetric microsensor was used to profile the concentration of total dissolved sulfide (HS- + H_2_S) throughout uninoculated S-plug tubes. To this end, dissolved sulfide (total of HS^−^ and H_2_S) in the S-plug medium was measured using cyclic voltammetry with the gold amalgam (Au/Hg) working microelectrode (Luther et al., 2003; Yücel, 2013). The three-electrode voltammetric sensor was calibrated for S(-II) before the first profile using standard additions of a stock solution prepared with Na_2_S and deoxygenated distilled water. In order to profile the sulfide concentration throughout the medium, the Au/Hg glass working electrode was attached to a micromanipulator with counter and reference electrodes placed in the overlying fluid of the plug. The working electrode was then vertically maneuvered with a minimum step of 1 mm. The scans were taken in cyclic voltammetry form, between −0.1 V and −1.8 V at a rate of 1000 mV sec^−1^. Before each scan, the electrode was electrochemically conditioned at −0.9 V for 10 s to remove any adsorbed species (Yücel, 2013). Detection limit was 0.2 μM for S(-II). Voltammograms were recorded from the electrodes using a bench-top potentiostat (PalmSens) and the data were processed with the software provided by the manufacturer.

## Results

### Composition of the Gas Emissions at Tor Caldara

The temperature measured during the summer in the water surrounding the Tor Caldara gas vents varied between 22 and 26°C, with an average salinity of 40 ppt (the avg. salinity of the Tyrrhenian Sea varies between 36 and 39 ppt) (Kallel et al., 1997). The pH (7.48) in the area of venting was slightly lower than the pH (7.9) away from venting. The venting gases were mainly composed of CO_2_ (avg. of 76.7 mol%) and H_2_S (avg. of 23.1 mol%) with minor contribution of CH_4_ (avg. of 0.18 mol%) and traces of CO (avg. of 0.008 mol%) and H_2_ (avg. of 0.001 mol%) (Table 1). Since the gas venting at Tor Caldara is a peripheral manifestation of the quiescent Colli Albani volcanic complex, whose activity began 600 ka ago (Carapezza et al., 2012), the system is thought to be very stable over time in comparison to the more dynamic venting associated with active hydrothermal vents.

**Table 1:** Gas composition at Tor Caldara shallow-water hydrothermal vent.

Gas composition in mol %					
Sample	H_2_S	H_2_	CO	CH_4_	CO_2_
1	13.8	0.001	0.005	0.21	86.0
2	33.4	0.001	0.011	0.17	66.4
3	37.2	0	0.006	0.16	62.6
4	30.3	0	0.006	0.19	69.5
5	15.7	0	0.023	0.22	84.1
6	27.4	0.001	0.006	0.19	72.4
7	15.4	0.001	0	0.18	84.5
8	17.5	0.002	0.024	0.17	82.3
9	18	0.002	0	0.20	81.8
10	22.7	0	0	0.15	77.2
Average	23.1	0.001	0.008	0.18	76.7
SD	7.9	0.0006	0.008	0.02	7.9

*SD, standard deviation.*

### Identification of Culturable Bacteria From Tor Caldara

**FIGURE 1:**
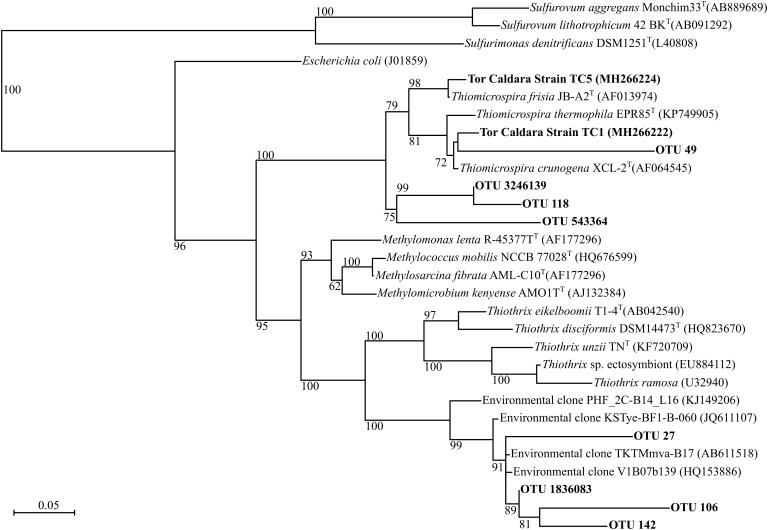
Maximum-likelihood phylogenetic tree derived from V4 regions and full length 16S rRNA gene sequences showing the positions of three *Thiothrix*-like CF-26 OTUs, three *Thiomicrospira* OTUs and *Thiomicrospira* strains TC1 and TC5 within the *Gammaproteobacteria*. aLRT branch support values higher than 50% were based on 1000 replicates and are shown at each node. Bar, 0.05% substitutions per position. Sequences belonging to the class *Epsilonproteobacteria* were used as outgroup.

**FIGURE 2:**
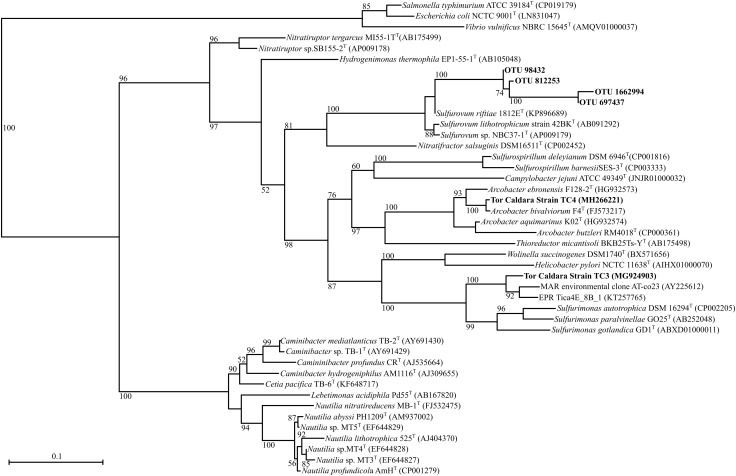
Maximum-likelihood phylogenetic tree derived from V4 regions and full length 16S rRNA gene sequences showing the position of OTUs classified as *Sulfurovum* within the *Epsilonproteobacteria* and strains TC3 and TC4. aLRT branch support values higher than 50% were based on 1000 replicates and are shown at each node. Bar, 0.1% substitutions per position. Sequences belonging to the class *Gammaproteobacteria* were used as outgroup.

EF samples were used as inoculum to obtain primary enrichments in a variety of heterotrophic and autotrophic media. Subsequently, pure cultures were obtained after undergoing three consecutive series of end-point dilutions. A total of 12 cultures were obtained from all the enrichments (Table 2). Among the isolates, four were sulfur-oxidizing chemolithotrophs: strain TC1 (Figure 1), whose 16S rRNA gene was 98.8% identical to that of *Thiomicrospira crunogena* (Jannasch et al., 1985), strain TC3 (92.8% identical to *Sulfurimonas gotlandica* Figure 2) (Labrenz et al., 2013), strains TC5 (100% identical to *Thiomicrospira frisia*; Figure 1) (Brinkhoff et al., 1999) and TC16 (98.1% similarity to *Sedimenticola thiotaurini*) (Flood et al., 2015). Phylogenetic analysis showed that the novel epsilonproteobacterial strain TC3 clustered together with two uncultured 16S rRNA gene clones recovered from deep-sea vents (Figure 2).

**Table 2:** Cultured strains from Tor Caldara and their closest relatives.

Strain	Enrichment origin	Class	Closest relative	16S rRNA gene % Identity	Medium
TC1	EF	*Gammaproteobacteria*	*Thiomicrospira crunogena*	98.8	DSMZ medium 1011 + O_2_/CO_2_ (liquid)
TC2	EF	*Gammaproteobacteria*	*Shewanella haliotis*	99.7	FS ASW (liquid)
TC3	EF	*Epsilonproteobacteria*	*Sulfurimonas gotlandica*	92.8	DSMZ medium 1011 +N_2_/CO_2_+ nitrate (liquid)
TC4	EF	*Epsilonproteobacteria*	*Arcobacter bivalviorum*	99.4	S plugs + Ferric ammonium citrate (solid)
TC5	EF	*Gammaproteobacteria*	*Thiomicrospira frisia*	100	Medium 1011 + O_2_/CO_2_ (liquid)
TC6	EF	*Gammaproteobacteria*	*Vibrio alginolyticus*	99.9	S plugs (solid)
TC10	EF	*Bacilli*	*Exiguobacterium profundum*	99.8	LS ASW (liquid)
TC12	EF	*Gammaproteobacteria*	*Marinobacter hydrocarbonoclasticus*	99.5	LS ASW (liquid)
TC13	EF	*Betaproteobacteria*	*Hydrogenaphaga taeniospiralis*	99.2	DSMZ medium 573 (solid)
TC14	EF	*Gammaproteobacteria*	*Agarivorans albus*	99.2	FS ASW (liquid)
TC15	EF	*Gammaproteobacteria*	*Halomonas aquamarina*	99.2	DSMZ medium 573 (solid)
TC16	EF	*Gammaproteobacteria*	*Sedimenticola thiotaurini*	98.1	DSMZ medium 1011 +N_2_/CO_2_+nitrate (liquid)

The remaining eight heterotrophic strains included: strain TC2 (99.7% identical to *Shewanella haliotis*) (Kim et al., 2007), strain TC4 (99.4% identity to *Arcobacter bivalviorum*; Figure 2) (Levican et al., 2012), strain TC6 (99.9 % identity to *Vibrio alginolyticus*) (Sakazaki, 1968), strain TC10 (99.8% identity to *Exiguobacterium profundum*) (Crapart et al., 2007), strain TC12 (99.5% identity to *Marinobacter hydrocarbonoclasticus*) (Gauthier et al., 1992), strain TC13 (99.2% identity to *Hydrogenophaga taeniospiralis*) (Lalucat et al., 1982; Willems et al., 1989), strain TC14 (99.2% identity to *Agarivorans albus*) (Kurahashi and Yokota, 2004) and strain TC15 (99.2% identity to *Halomonas aquamarina;* Dobson and Franzmann, 1996). Strain TC13 grew either chemolithoautrophically using sodium sulfide or heterotrophically using acetate.

### Microscopy

Scanning electron micrographs of the EF showed at least three types of different filaments: thin filaments with sulfur inclusions (Supplementary Figure 2a), thicker filaments with an outer sheath and segmented cells (Supplementary Figure 2b) and *Thiothrix*-like sheathed cells with visible septa (Supplementary Figure 2c). The YF filaments appeared to be embedded in a matrix containing sulfur crystals (Supplementary Figure 2d). The crystals were identified by using the elemental analyzer in conjunction with the SEM.

### Composition of Tor Caldara Microbial Communities

A total of 1,311,749 sequences were generated, which clustered in 19,128 unique OTUs at 97% similarity across the 16 samples. Out of these, 19,100 OTUs were bacterial and only 28 OTUs were archaeal. Archaeal sequences contributed to less than 0.01% of the total sequences. Rarefaction curve analysis showed that, on average, EF community was more diverse than the YF (Supplementary Figure 3). The total EF community was more diverse than its active fraction (avg. chao1 of 5412 vs. 3909; Supplementary Table 2), while the total and active fraction of YF showed comparable diversity (avg. chao1 of 4111 and 4621, respectively; Supplementary Table 2).

### Established and Young Filamentous Microbial Communities

Overall, 15 phyla were abundant (≥0.1%) in the EF community, as opposed to 8 phyla in the YF community (Supplementary Table 3).

**FIGURE 3:**
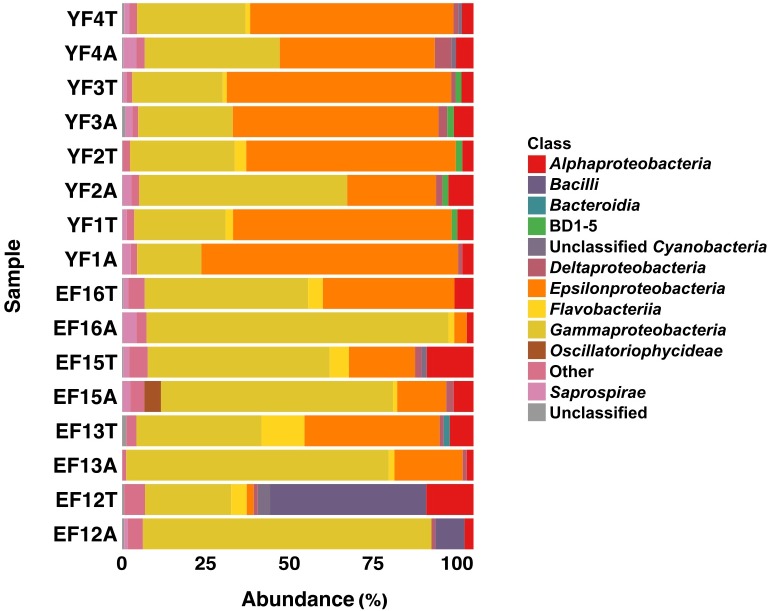
Class level distribution of 16S rRNA gene and transcript sequences recovered from EF and YF. Phylogenetic categories representing classes that account for at least 1% of overall abundance in all samples are shown. YF, Young Filaments; EF, Established Filaments; T, Total community (rRNA genes); A, Active Community (rRNA transcripts); the number for YF denotes the slide replicate, the number for EF denotes the sampling year.

The total and active fractions of the EF community were numerically dominated by *Proteobacteria* (75.3 and 91.2% on average, respectively), followed by *Bacteroidetes* (9.2 and 4.1%, respectively) and *Cyanobacteria* (2.3% each). The 2012 filament sample (EF12) was an exception, with *Firmicutes* making up 45.2 and 8.3% of the total (EF12T) and active filament (EF12A) community. The total and active fractions of the YF community were numerically dominated by *Proteobacteria* (93.7 and 94% on average, respectively), followed by *Bacteroidetes* (3.5% each) and candidate phylum GN02, later renamed as *Gracilibacteria* (Rinke et al., 2013) (1.5 and 1.4%, respectively). Within the *Proteobacteria*, the classes *Gammaproteobacteria* and *Epsilonproteobacteria* dominated both the EF and the YF communities, respectively (Figure 3).

**FIGURE 4:**
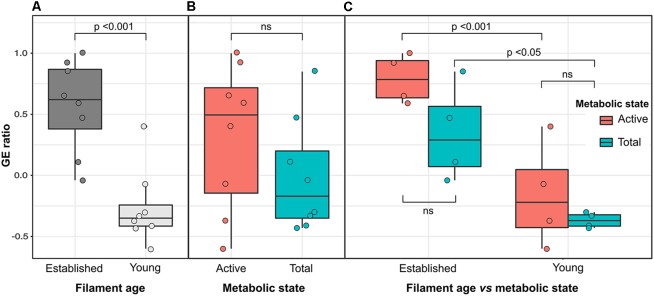
Variation of the *Gamma*- to *Epsilonproteobacteria* ratio (GE ratio) between the established (EF) and young filament (YF) communities. **(A)** Total plus active established vs. young filaments ratio; **(B)** Active vs. total ratio for established and young filaments combined; **(C)** Active and total community ratio calculated independently for the established and young filament communities.

On average, *Gammaproteobacteria* constituted 39.5% of the total EF community and 77.1% of its active fraction, whereas *Epsilonproteobacteria* constituted 24.2% of the total EF community and 12.3% of its active fraction. A reverse trend was observed in the YF community where, on average, *Epsilonproteobacteria* constituted 60.9% of the total filament community and 50.3% of its active fraction, whereas *Gammaproteobacteria* constituted 28.1% of the total filament community and 35.7% of its active fraction. The *Gammaproteobacteria* to *Epsilonproteobacteria* ratio (GE ratio) shows statistically significant differences between the established vs. young filaments (ANOVA *p* < 0.001; Figure 4A) with a predominance of *Gammaproteobacteria* in the established filaments (GE ratio of 0.57 ± 0.38) and a predominance of *Epsilonproteobacteria* in the young filaments (GE ratio of −0.26 ± 0.31). This difference is not evident when the GE ratio of the active vs. total community is calculated (ANOVA *p* > 0.05; Figure 4B), suggesting that the shift in the community is controlled predominantly by the age rather than by the metabolic state of the communities. Further, both the active community and the total community are statistically different between the established and young filaments (Tukey HSD *post hoc* test *p* < 0.01 for the active and *p* < 0.05 for the total community respectively; Figure 4C).

The other abundant groups in the total and active fractions of the EF community were *Alphaproteobacteria* (9.7 and 3%, respectively), *Flavobacteria* (6.6 and 1.5 %, respectively), *Saprospirae* (2.3 and 1.5%, respectively) and *Deltaproteobacteria* (1.4 and 1.5%, respectively) (Figure 3). The remaining fractions of the total and active YF community were *Alphaproteobacteria* (3.6 and 5.2%, respectively), *Flavobacteriia* (1.9% and undetectable, respectively), *Saprospirae* (1.2 and 2.6%, respectively) and *Deltaproteobacteria* (1.2 and 2.5%, respectively) except for the BD1-5 group (1.7 and 1.7%, respectively) within the candidate phylum GN02/*Gracilibacteria*, which was unique to the YF community.

**FIGURE 5:**
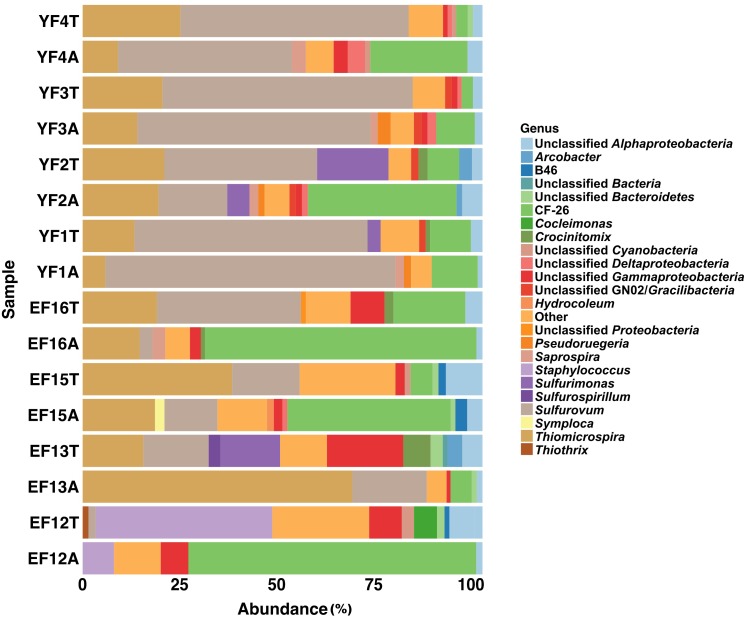
Genus level distribution of 16S rRNA gene and transcript sequences recovered from EF and YF samples. Phylogenetic categories representing genera that account for at least 1% of overall abundance in all samples are shown. YF, Young Filaments; EF, Established Filaments; T, Total community (rRNA genes); A, Active Community (rRNA transcripts); the number for YF denotes the slide replicate, the number for EF denotes the sampling year.

At the genus level, the most abundant members of the total EF community were related to the sulfur-oxidizing *Gammaproteobacteria*, *Thiomicrospira*, *Thiothrix*, *Cocleimonas*, *Thiothrix*-related CF-26, and to the *Epsilonproteobacteria*, *Sulfurovum*, and *Sulfurimonas* (Figures 1, 2, 5). Many sequences within the *Gammaproteopbacteria* that were not resolved at the genus level were manually analyzed and were closely related (94–96% similarity) to *Thioprofundiculum*, *Thiothrix*, *Thiohalomonas*, *Thiomicrospira*, and *Galenea*. The *Alphaproteobacteria*-related sequences were mainly affiliated with the *Rhodobacteraceae* order. Sequences in the *Bacteriodetes* phylum were mainly related to the *Lutibacter*, *Crocinitomix*, and *Saprospira* genera (Figure 5). In spite of this diversity in the total community, *Thiomicrospira* and *Thiothrix*-related CF-26 heavily dominated the active EF community, with the latter accounting for more than 60% of the sequences in three out of the four samples (Figure 5). In the total YF community, *Sulfurovum*, *Thiomicrospira*, *Thiothrix*-related CF-26 and *Sulfurimonas* were the most abundant genera, with *Sulfurovum* (53.7% on average) dominating the community (Figure 5). Sequences related to *Crocinitomix* and *Saprospira* within the *Bacteriodetes* phylum were also recovered in addition to unresolved sequences belonging to *Deltaproteobacteria* and the order *Rhodobacteraceae*. *Sulfurovum* (57.6%) also dominated the active fractions of all, but one of the YF communities.

### Adaptation of *Sufurovum* and *Thimicrospira* spp. to Different Sulfide Concentrations

**FIGURE 6:**
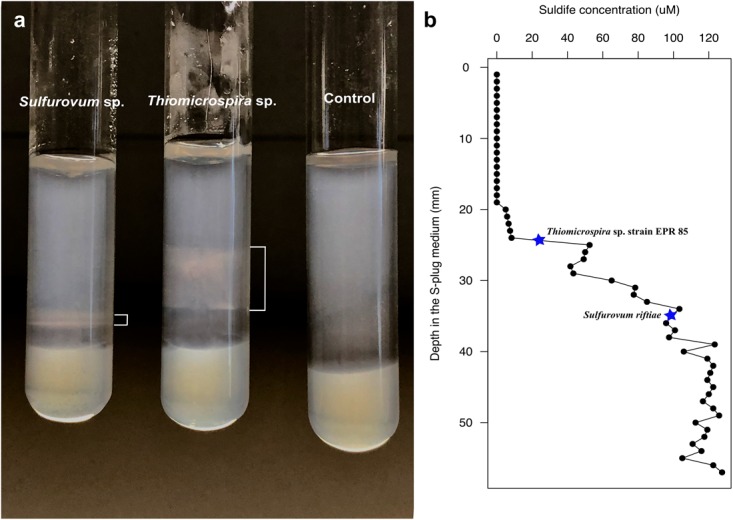
Growth of *Sulfurovum riftiae* and *Thiomicrospira* sp. strain 85 in sulfide gradient medium; **(a)** growth pattern in S-plug tubes; **(b)** Total sulfide concentration along the gradient. Stars indicate the total sulfide concentration for optimal growth of the two strains.

Since phylotypes related to sulfur-oxidizing *Sulfurovum* (*Epsilonproteobacteria*) dominated the YF, while *Thiomicrospira* and *Thiothrix* (*Gammaproteobacteria*) dominated the EF (Figure 5), we posited that the observed shift might be related, among other factors, to a different adaptation to sulfide of the two groups of bacteria. To test this hypothesis, we grew *Sulfurovum riftiae* (*Epsilonproteobacteria*; Figure 2) and *Thiomicrospira* sp. EPR 85 (*Gammaproteobacteria*; Figure 1) from the laboratory culture collection in S-plug medium. These two strains were chosen for the following reasons: (1) both bacteria are closely related to strains or phylotypes obtained from the Tor Caldara biofilms (Figures 1, 2); (2) the *Thiomicrospira* strains isolated from Tor Caldara were not characterized beyond the 16S rRNA gene sequence, and generally much harder to maintain in culture; (3) in contrast, *Thiomicrospira* sp. strain EPR 85 and *Sulfurovum riftiae* are well characterized physiologically and their genomes have been sequenced; (4) to date, no *Sulfurovum* spp. have been isolated from shallow-water marine geothermal systems. The sulfide gradient that forms in the S-plug tubes allows the bacteria to position themselves at their optimal sulfide concentration. Subsequently, we used cyclic voltammetry to profile the sulfide concentration throughout the medium and we evaluated the growth of the two strains relative to the concentration of sulfide. *Thiomicrospira* sp. EPR 85 grew over a relatively broad range of sulfide concentrations: while most of the biomass was visible at about 20–25 mm from the top of the agar, where the sulfide concentration varied between 5 and 52.5 μM (Figures 6a,b), some growth could be detected down to 32 mm, where the sulfide concentration was 77.5 μM (Figures 6a,b). In contrast, *S. riftiae* showed a much more restricted growth pattern with a band at about 35 mm from the top of the agar, where the sulfide concentration was about 100 μM (Figures 6a,b).

## Discussion

The aim of this study was to explore the microbial diversity and gas composition of the newly discovered shallow-water gas vents located at Tor Caldara, Italy. The venting gas is mainly composed of CO_2_ (avg. 77 mol%) and H_2_S (avg. 23 mol%), with trace amounts of CH_4_ (0.18 mol%). This is in accordance with observations at other shallow-water vents in the Tyrrhenian Sea and elsewhere (Dando et al., 1999; Gugliandolo et al., 1999; Tarasov et al., 2005; Maugeri et al., 2008). However, the Tor Caldara gas is more enriched in H_2_S as compared to other shallow-water vent sites. This could be attributed to the fact that, unlike other shallow-water vent sites, the temperature at Tor Caldara is not affected by geothermal processes and H_2_S is a product of low temperature hydrothermal activity in the perivolcanic outgassing areas (Pizzino et al., 2002; Boatta et al., 2013). Salinity of the Mediterranean is known to be high (between 36 ppt near Gibraltar to 39 ppt in the eastern basin) because the evaporation processes are more dominant than fresh water input from precipitation and river discharge (Kallel et al., 1997). At 40 ppt, salinity at Tor Caldara was found to be marginally higher that the average salinity in the Mediterranean Sea. The pH was slightly lower in the vicinity of the venting due to the high input of CO_2_ from the gas emissions.

In the course of several summers of sampling the Tor Caldara gas vents, we noted that the density of the sessile filamentous biofilms was substantially lower following intense wave motion due to high winds. This was predictable, as shallow-water vents are dynamic environments, strongly influenced by factors such as tides, winds and waves (Yücel et al., 2013; Price and Giovannelli, 2017), and implies that these biofilm communities must be experiencing intermittent disruption, resetting the clock for new colonization.

We investigated the total and active fractions of the microbial biofilm communities using 16S rRNA gene and transcript amplicon analysis. Hierarchical clustering (Supplementary Figure 4) and nMDS (Supplementary Figure 5) analyses carried on the microbial biofilm communities showed that, with few exceptions, EF and YF communities clustered separately. One such exception is EF12T, in which *Firmicutes* comprised 45.2% of the OTUs (Figures 3, 5 and Supplementary Figure 4). We tend to rule out a possible laboratory contamination for two reasons: (1) The no-template control associated with this sample did not show amplification; (2) the abundance of the *Firmicutes* in the corresponding active fraction (EF12A sample) was much lower and showed that the main active bacteria were related, similarly to the other established filaments, to the *Thiothrix*-related CF-26 group (Figures 3, 5). One possible explanation for the abundance of *Firmicutes* in EF12T is that the rock substrate and sand associated with the filaments were co-sampled in 2012. This was the first year of sampling and, instead of collecting the filaments with a syringe, we scraped them from the substrate, with possible carryover of sedimentary debris. The presence of *Firmicutes* in the Tor Caldara sediments is not surprising, as these bacteria have been found to be relatively abundant in coastal sediments associated with gas venting (Kerfahi et al., 2014).

### Succession in Microbial Communities of the Young and Established Filaments

Microbial biofilms have been studied at cold seeps (Ristova et al., 2015; Paul et al., 2017), sulfidic caves (Engel et al., 2004; Macalady et al., 2006), thermal springs (Coman et al., 2013; Beam et al., 2016), mud volcanoes (Heijs et al. (2005); Omoregie et al., 2008), as well as deep-sea (Lopez-Garcia et al., 2003; Alain et al., 2004; Crépeau et al., 2011; Dahle et al., 2013; Gulmann et al., 2015; O’Brien et al., 2015; Stokke et al., 2015; Teske et al., 2016) and shallow-water vents (Gugliandolo et al., 2006; Miranda et al., 2016). Many of these microbial communities are dominated by members of the *Gammaproteobacteria* and *Epsilonproteobacteria* (Macalady et al., 2006; Crépeau et al., 2011; O’Brien et al., 2015). However, there is a paucity of such studies in the Mediterranean Sea, where previous work has focused more on sedimentary environments (Gugliandolo and Maugeri, 1993; Maugeri et al., 2013; Kerfahi et al., 2014).

Previous field observations of the distribution of microorganisms in biofilms from geothermal habitats indicated that *Epsilon*- and *Gammaproteobacteria* are spatially segregated, with the former dominating higher sulfide habitats and the latter being more prevalent in lower sulfide, possibly more oxic habitats (Engel et al., 2004; Reigstad et al., 2011; Giovannelli et al., 2013; O’Brien et al., 2015; Miranda et al., 2016). Based on these observations, and on the high concentrations of sulfide measured in the gases at Tor Caldara (Table 1), we had predicted that the EF biofilms would be dominated by sulfide-oxidizing *Epsilonproteobacteria*. However, in the initial stages of this study, we detected a prevalence of *Gammaproteobacteria* related to the genera *Thiothtrix* and *Thimicrospira* in the EF biofilms (Figures 1, 5). Following these initial observations, we hypothesized that sulfide-oxidizing *Epsilonproteobacteria* might play a role in the early stages of biofilm formation at Tor Caldara, and that a colonization pattern might occur on a temporal, rather than spatial, scale. To test this hypothesis, in 2016 we conducted a biofilm colonization experiment by deploying sterile glass slides in the same area for 4 days, till visible biofilm (YF) formation was observed (Supplementary Figure 1b). Then we analyzed the taxonomic composition of the YF and we compared that with that of the EF biofilms (Supplementary Figure 1a). Both types of filaments showed similar diversity at the phylum level, except for *Cyanobacteria* and GN02/*Gracilibacteria*, which were unique to the EF and YF, respectively. Members of candidate phylum GN02/*Gracilibacteria* were first reported from a high sulfide concentration zone of a hyper saline microbial mat in Mexico and, later, single amplified genomes (SAGs) from this phylum were recovered from the East Pacific Rise hydrothermal vent system (Ley et al., 2006; Rinke et al., 2013). The presence of *Cyanobacteria*-related sequences in both the total and active fraction of established filaments at Tor Caldara might indicate a more permissible (e.g., less sulfidic and more oxic) environment as compared to the newly formed community (Ley et al., 2006).

The white and feathery EF resembled those seen at the oxic/anoxic boundary of sulfidic environments. Members of the *Thiotrichales*, especially those closely related to *Thiothrix*, have been shown to dominate these white filaments (Engel et al., 2004; Gugliandolo et al., 2006; Macalady et al., 2006; Reigstad et al., 2011; O’Brien et al., 2015). We observed sequences related to *Thiothrix* in our EF samples, where *Thiomicrospira* and *Thiothrix*-related CF-26 dominated the total and active fraction of these communities. Two species of *Thiomicrospira*, *T. frisia* and *T. crunogena* were also isolated from EF in pure culture under strict sulfur-oxidizing chemolithoautotrophic conditions, further establishing their abundance and importance. *Thiothrix*-related CF-26 is an uncultured genus first described as a clone recovered from the flocculent mats associated with submarine volcanoes along the Kermadec Arc. The OTUs annotated as CF-26 show high 16S rRNA sequence identity (>97%) to sequences recovered from Hatoma Knoll deep-sea vent site (GenBank accession number AB611518) (Yoshida-Takashima et al., 2012), microbial mats at shallow-vents from South Tonga Arc (GenBank accession number HQ153886) (Murdock et al., 2010) and Kuieshan Island shallow water vents (GenBank accession number JQ611107) (unpublished) with *Thiothrix* as the closest genus (16S rRNA gene sequence identity: 88–90%; Figure 1). However, the scanning electron micrographs of the filaments didn’t show rosette-like formations that are characteristic of *Thiotrix*, while sheathed cells with visible septa as described by Williams et al. (1987) were observed along with other types of filaments (Supplementary Figure 2). Members of the genus *Thiothrix* are sulfur-oxidizing chemoautotrophic, heterotrophic as well as mixotrophic bacteria (Howarth et al., 1999). Similar to the other chemoautotrophic members of the *Gammaproteobacteria*, *Thiotrix* species fix CO_2_ via the CBB cycle (Odintsova et al., 1993). Unfortunately, our attempts to cultivate *Thiotrix*-related bacteria in selective medium were unsuccessful. *Thiomicrospira* are also sulfur oxidizing obligate chemolithoautotrophs that fix CO_2_ using the CBB cycle, with mixotrophy observed in some species (Boden et al., 2017). Sequences related to the genus B46 within *Gammaproteobacteria* with 95% similarity to *Methylomolas* and *Methylococcaceae* were also recovered. Similar sequences have been recovered from the Tica vent on the East Pacific Rise deep-sea hydrothermal vent system (GenBank accession number KT257824) (Gulmann et al., 2015) as well as microbial mats at White Point, California (GenBank accession number KX422153) (Miranda et al., 2016) indicating potential occurrence of methylotrophy in addition to chemoautotrophy. Successful isolation of *Sedimenticola thiotaurini*, another sulfur-oxidizing chemolithotroph belonging to *Gammaproteobacteria*, further confirmed their dominance in the EF. The isolation of several non-sulfur-oxidizing heterotrophic bacterial strains suggests that the EF is a complex community that also harbors heterotrophs, in addition to primary producers. The enrichment and isolation approach provided an initial culture collection from the Tor Caldara gas vents for future physiological and genomic characterization (Patwardhan and Vetriani, 2016).

Following a 4-day deployment, the glass slides (YF) were predominantly colonized by *Sulfurovum* species. Sequences related to the genus *Sulfurospirillum* were also recovered. Both genera are sulfur-oxidizing chemolithoautotrophic members of *Epsilonproteobacteria* that are abundant in both shallow and deep-sea vents environments (Sievert and Vetriani, 2012; Giovannelli et al., 2013; Vetriani et al., 2014; O’Brien et al., 2015; Miranda et al., 2016). We also isolated a pure culture from Tor Caldara that had ∼93% 16S rRNA gene sequence similarity to *Sulfurimonas gotlandica*, which is another sulfur-oxidizing chemolithoautotrophic *Epsilonproteobacterium*.

### Adaptation to Different Sulfide Concentrations May Drive Ecological Succession of *Epsilon*- and *Gammaproteobacteria* in Young and Established Biofilms

The dominance of *Epsilonproteobacteria* related to *Sulfurovum* spp. in the YF active community indicates that these bacteria were the early colonizers in the Tor Caldara filamentous biofilms and that *Gammaproteobacteria* related to *Thiomicrospira* and *Thiothrix* spp. became prevalent as the biofilm matured (Figures 3, 5). Therefore, we posited that the metabolic activities of *Sulfurovum* sp. during the early stages of colonization may be critical to condition the environment for the settlement of subsequent species. Previous studies have observed that *Epsilon*- and *Gammaproteobacteria* occupy spatially separated niches based on their adaptation to sulfide concentrations (Engel et al., 2004; Reigstad et al., 2011; O’Brien et al., 2015; Miranda et al., 2016; Meier et al., 2017). In particular, in a 16S rRNA gene- and metagenomic-based study of the distribution of microorganisms at deep-sea hydrothermal vents in the Manus Basin, off Papua New Guinea, Meier et al. (2017) proposed that sulfide and oxygen concentrations are determining factors for niche partitioning between sulfur-oxidizing *Epsilon*- and *Gammaproteobacteria*. However, to our knowledge this hypothesis had not been experimentally tested prior to this study.

We tested this hypothesis experimentally by evaluating the growth pattern of an *Epsilonproteobacterium* (*S. riftiae*) and a *Gammaproteobacterium* (*Thiomicrospira* sp. EPR 85) relative to the concentration of total dissolved sulfide throughout S-plug tubes. Our findings show that *S. riftiae* grew at higher sulfide concentration (100 μM) than *Thiomicrospira* sp. EPR 85 (5 – 52.5 μM; Figures 6a,b). These sulfide concentrations are consistent with those measured *in situ* at deep-sea hydrothermal vents. For instance, the sulfide concentrations measured by O’Brien et al. (2015) in *Epsilonproteobacteria*-dominated diffuse flow deep-sea vents were up to 67 mM, while it ranged between 0.3–2.9 mM at *Gammaproteobacteria*-dominated adjacent control sites. Similarly, Gulmann et al. (2015) reported maximum dissolved sulfide concentrations of 100 μM in fluids from a diffuse flow vent at the same site. It is worth noting that Meier et al. (2017) inferred that the distribution of SUP05-related *Gammaproteobacteria* at deep-sea vents correlated with sulfide concentrations between 0 and 100 μM, while *Sulfurovum* and *Sulfurimonas*-related *Epsilonproteobacteria* correlated with sulfide concentrations between about 100 and 750 μM. While some of the latter concentrations are higher than those measured in the S-plug experiment, it should be noted that *S. riftiae* grew near the highest sulfide concentration attainable in the S-plug tube. Hence, it is possible that its observed narrow growth range could be limited by sulfide availability and that the bacterium is able to tolerate higher sulfide concentrations than those observed in this experiment.

Based on these observations, it is reasonable to infer that, at the early stages of formation of the Tor Caldara biofilms, *Sulfurovum*-related spp. experience and cope with the high sulfide (and possibly low oxygen) concentration in the gas emissions. Laboratory studies of pure cultures of *Sulfurovum* sp. revealed that these bacteria conserve energy by the oxidation of reduced sulfur species to sulfate (Yamamoto et al., 2010). Hence, at Tor Caldara, respiration by *Sulfurovum* spp. and other sulfur-oxidizing *Epsilonproteobacteria* may contribute to the decrease in sulfide concentration within the biofilm structure during the early stages of colonization, effectively conditioning the habitat via sulfide detoxification for the subsequent colonization by *Gammaproteobacteria* related to *Thiomicrospira* and *Thiothrix* spp. This is consistent with the observation that the less sulfide-tolerant, aerobic *Gammaproteobacteria* become dominant in the EF community (Figures 3, 5). Interestingly, the observed temporal succession from *Epsilon*- to *Gammaprotebacteria* dominance in the Tor Caldara biofilms appears to be driven by ecosystem conditioning rather than by the spatial segregation often observed at deep-sea hydrothermal vents (Engel et al., 2004; Reigstad et al., 2011; O’Brien et al., 2015; Miranda et al., 2016; Meier et al., 2017).

## Conclusion

Our findings show that the gas composition of the Tor Caldara shallow-water vents is composed mainly by CO_2_ and H_2_S. These gases support dense sessile filamentous microbial biofilms prevalently composed of sulfur-oxidizing *Epsilon*- and *Gammaproteobacteria*. We observed a temporal succession in the biofilm species composition that appears to be driven, among other factors, by the different adaptation of some of the most abundant *Epsilon*- and *Gammaproteobacteria* (*Sulfurovum* and *Thiomicrospira* spp.) to sulfide concentration. Future studies investigating the metabolic potential and function of these biofilm communities will be important to further elucidate their ecophysiological role.

## Author Contributions

SP performed the experiments, analyzed the data, and wrote the manuscript. DF analyzed the data and participated in the writing of the manuscript. DG collected samples, analyzed the data, and participated in the writing of the manuscript. MY analyzed the data and participated in the writing of the manuscript. CV directed and supervised the research, collected samples, and wrote the manuscript.

## Conflict of Interest Statement

The authors declare that the research was conducted in the absence of any commercial or financial relationships that could be construed as a potential conflict of interest.
