# Supplementary material for: Ecological Succession of Sulfur-Oxidizing Epsilon- and Gammaproteobacteria During Colonization of a Shallow-Water Gas Vent
Source: Front Microbiol. 2018 Dec 6;9:2970. doi: 10.3389/fmicb.2018.02970 (PMC6291522; doi:10.3389/fmicb.2018.02970)
Supplement: Supplementary file 1 [file Data_Sheet_1.docx]

Supplementary Material

Supplementary Table 1. Percent relative abundances of *Gammaproteobacteria* and *Epsilonproteobacteria* and GE ratio

| Sample | Relative Abundance (%) | | GE ratio |
| --- | --- | --- | --- |
|  | *Gammaproteobacteria* | *Epsilonproteobacteria* | |
| EF12A | 82.12 | 0.007 | 1 |
| EF13A | 74.71 | 19.41 | 0.59 |
| EF15A | 66.04 | 13.95 | 0.65 |
| EF16A | 85.93 | 3.59 | 0.92 |
| EF12T | 24.5 | 2.02 | 0.85 |
| EF13T | 35.57 | 38.44 | -0.04 |
| EF15T | 51.75 | 18.81 | 0.47 |
| EF16T | 46.47 | 37.46 | 0.11 |
| YF1A | 18.28 | 73.04 | -0.6 |
| YF2A | 59.24 | 25.29 | 0.4 |
| YF3A | 26.9 | 58.56 | -0.37 |
| YF4A | 38.39 | 44.14 | -0.07 |
| YF1T | 25.98 | 62.19 | -0.41 |
| YF2T | 29.83 | 59.66 | -0.33 |
| YF3T | 25.76 | 63.84 | -0.43 |
| YF4T | 30.9 | 57.88 | -0.3 |

Supplementary Table 2. Shannon and Chao1 indices

| **Type** | **Avg. Chao1 (Richness)** | **Avg. Shannon (Diversity)** |
| --- | --- | --- |
| Active Established Filaments | 3909.11 | 4.16 |
| Total Established Filaments | 5412.50 | 6.58 |
| Active Young Filaments | 4621.15 | 5.11 |
| Total Young Filaments | 4111.51 | 5.26 |

Supplementary Table 3. Phyla with relative abundance above 0.1 % in EF and YF 16S rRNA gene and transcript libraries. All abundances are averages of all samples except for the ones in blue which were found only in single samples.

|  | **Young Filaments (YF)** | | **Established Filaments (EF)** | |
| --- | --- | --- | --- | --- |
| **Phylum** | **Total** | **Active** | **Total** | **Active** |
| Above 1% | |  |  |  |
| *Proteobacteria* | 94.0% | 93.7% | 75.3% | 91.2% |
| *Firmicutes* | nd | nd | 45.2% | 8.3% |
| *Bacteroidetes* | 3.5% | 3.5% | 9.2% | 4.1% |
| *Cyanobacteria* | 0.5% | 0.6% | 2.3% | 2.3% |
| GN02/*Gracilibacteria* | 1.5% | 1.6% | 0.6% | 0.4% |
|  |  |  |  |  |
| Between 0.1 and 1% | |  |  |  |
| *Acidobacteria* | nd | 0.19% | 0.18% | nd |
| *Actinobacteria* | 0.10% | nd | 0.12% | 0.14% |
| *Chloroflexi* | nd | nd | 0.19% | nd |
| *Parvarchaeota* | nd | nd | 0.11% | nd |
| *Planctomycetes* | 0.13% | nd | 0.39% | 0.17% |
| *Spirochaetes* | nd | nd | 0.10% | nd |
| *Tenericutes* | nd | nd | 0.23% | nd |
| Unc. *Bacteria* | 0.29% | 0.37% | 0.61% | 0.32% |
| *Verrucomicrobia* | 0.10% | nd | 0.23% | 0.10% |

Supplementary Fig. 1. a, Established filaments (EF); b, Young filaments (YF).


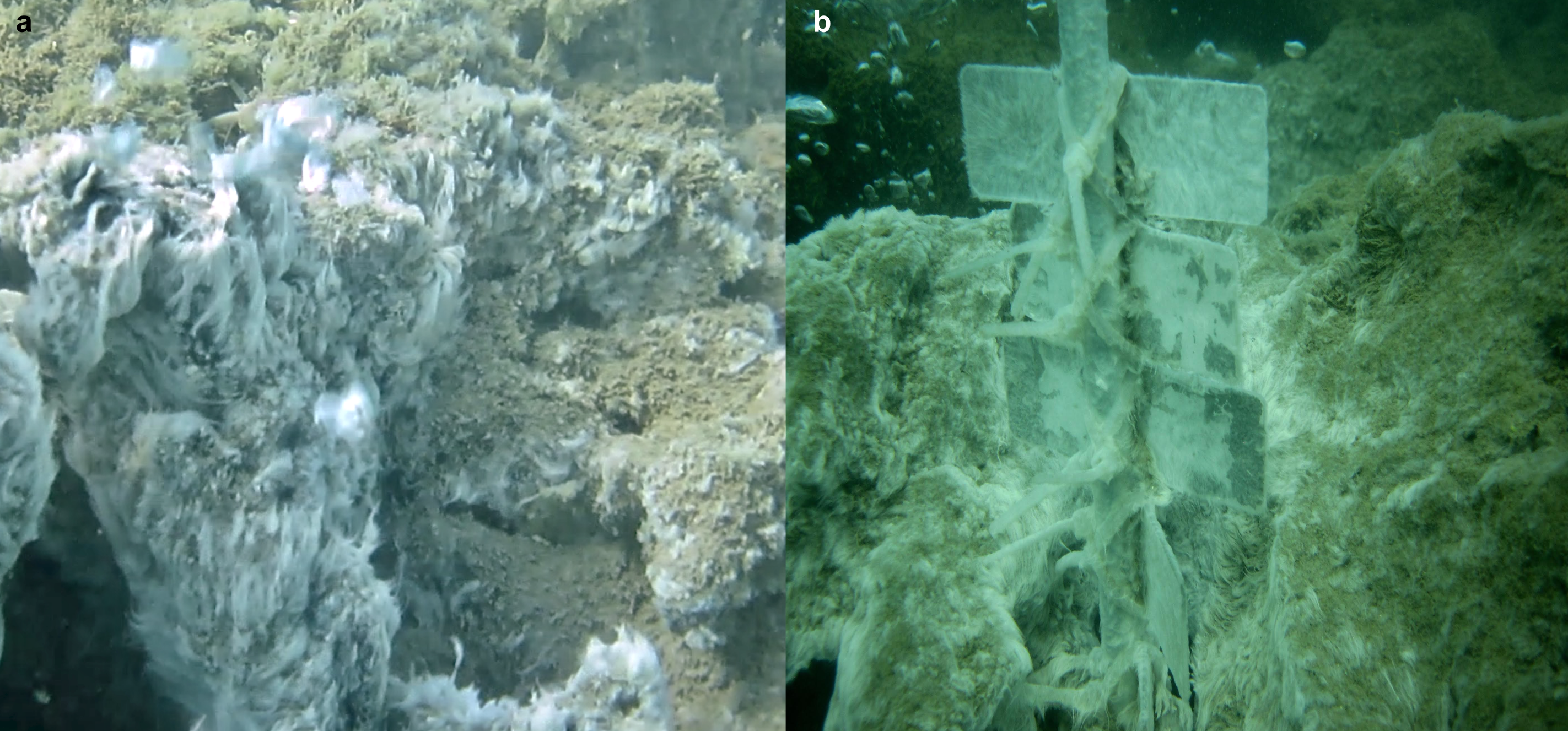


Supplementary Fig. 2. Scanning electron micrographs of EF (a, b, c) & YF (d): a: thin filaments with sulfur inclusion as indicated by the arrows, b: thick filaments with outer sheath and segments cells, c: *Thiothrix*-like filament, d: filamentous matrix with embedded sulfur crystals. Bars: 10 μm (a), 30 μm (b), 30 μm (c), 30 μm (d).


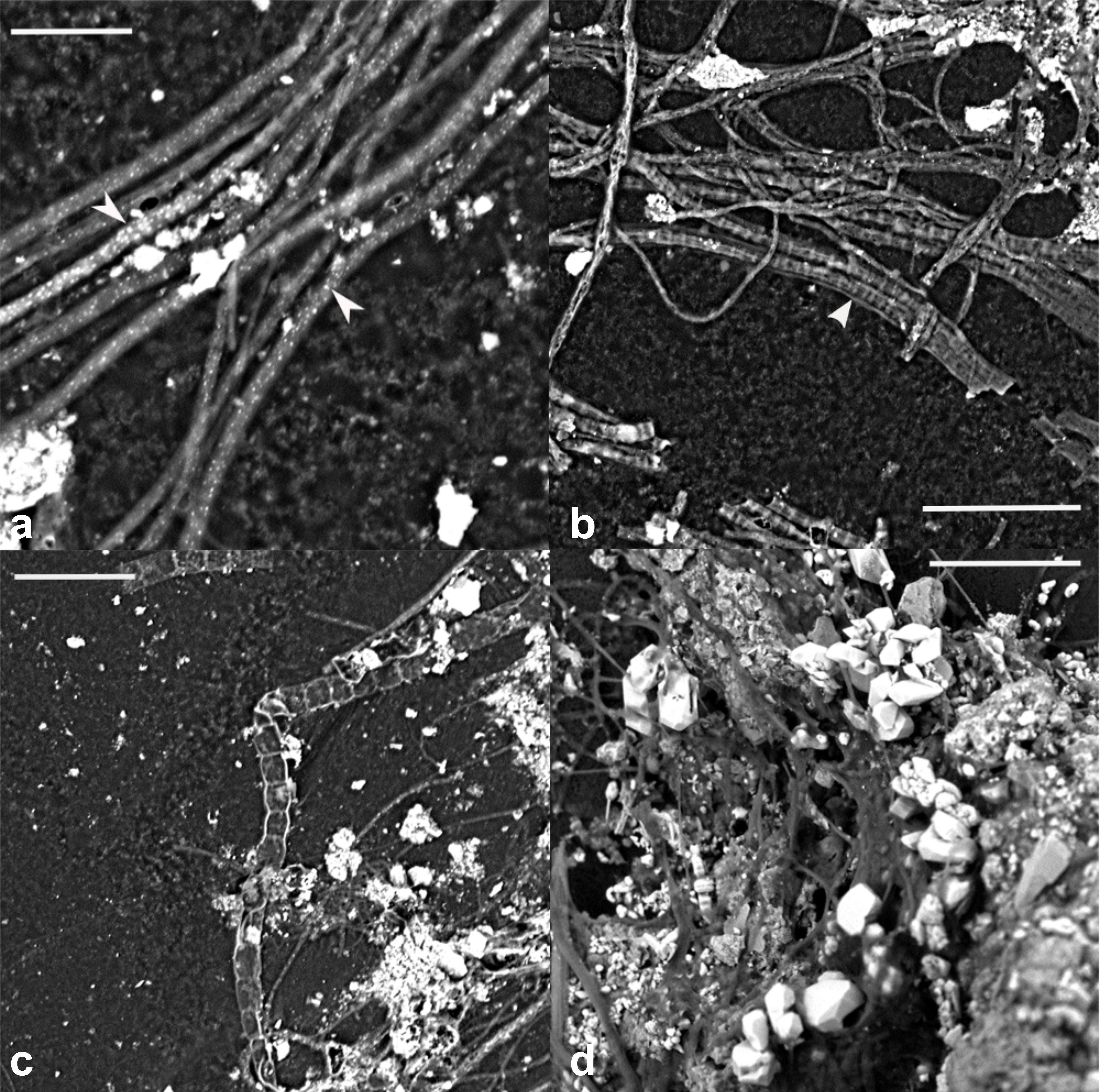


**Bioinformatic analyses**

Alpha and beta diversity estimates were calculated using the respective QIIME scripts: alpha_diversity.py and beta_diversity_through_plots.py. The weighted UniFrac distance matrix computed using the beta diversity script was used to perform non-metric multi-dimensional scaling (nMDS) using the nmds.py script. The distance matrix was also imported into R 3.4.3 to perform cluster analysis using the ward method [1].

Supplementary Fig. 3. Avg. rarefaction curve for 16S rRNA gene and transcript sequences


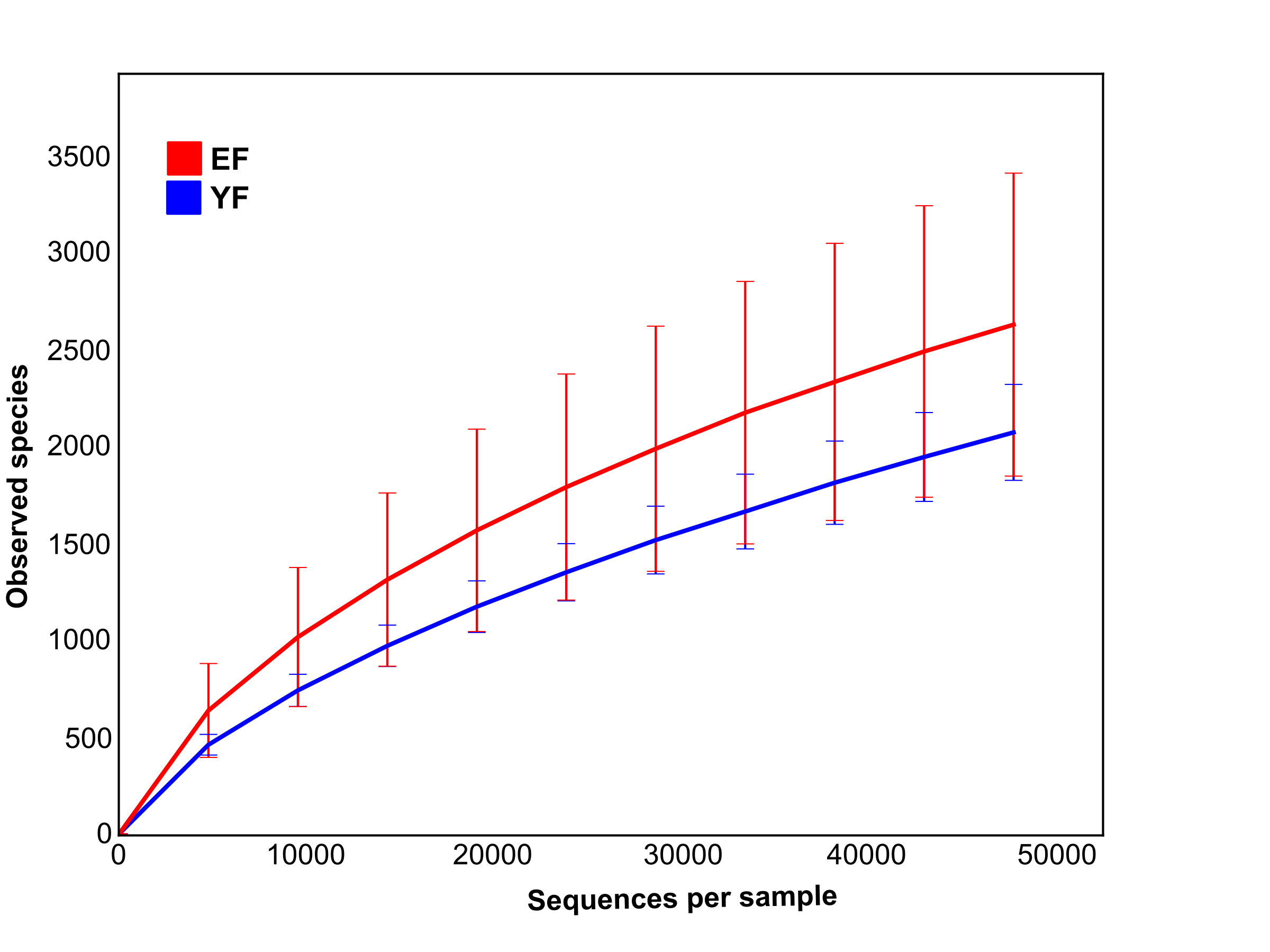


Supplementary Fig. 4. Results of the hierarchical clustering analysis based on 16S rRNA gene and transcript phylogenies and frequencies in the different samples. YF: Young Filaments; EF: Established Filaments; T: Total community (rRNA genes); A: Active Community (rRNA transcripts); the number for YF denotes the slide replicate, the number for EF denotes the sampling year as presented in Supplementary Table 1.


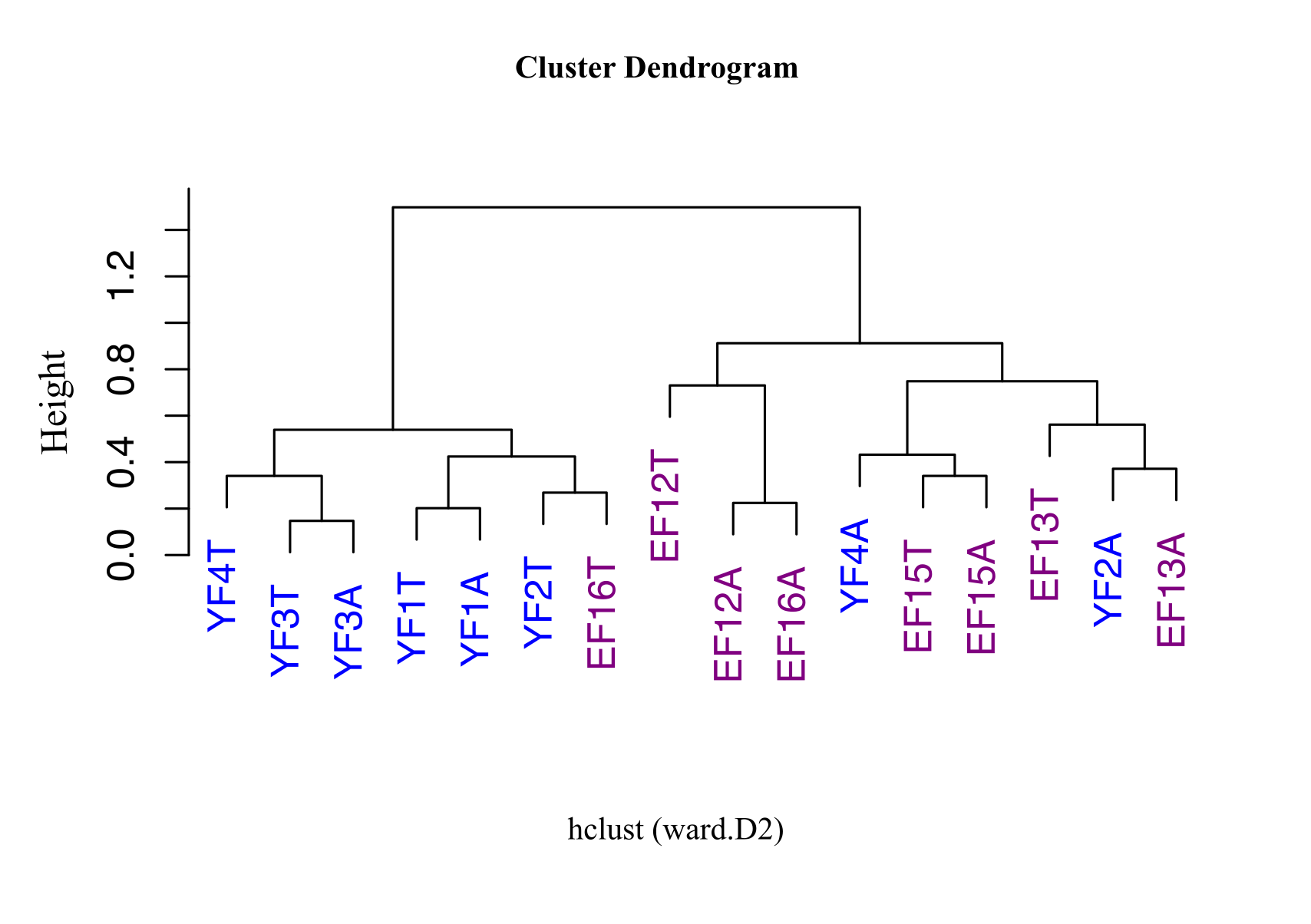


Supplementary Fig. 5. Results of the nMDS analysis based on 16S rRNA gene and transcript phylogenies and frequencies in the different filament type. YF: Young Filaments; EF: Established Filaments; T: Total community (rRNA genes); A: Active Community (rRNA transcripts); the number for YF denotes the slide replicate, the number for EF denotes the sampling year as presented in Supplementary Table 1.


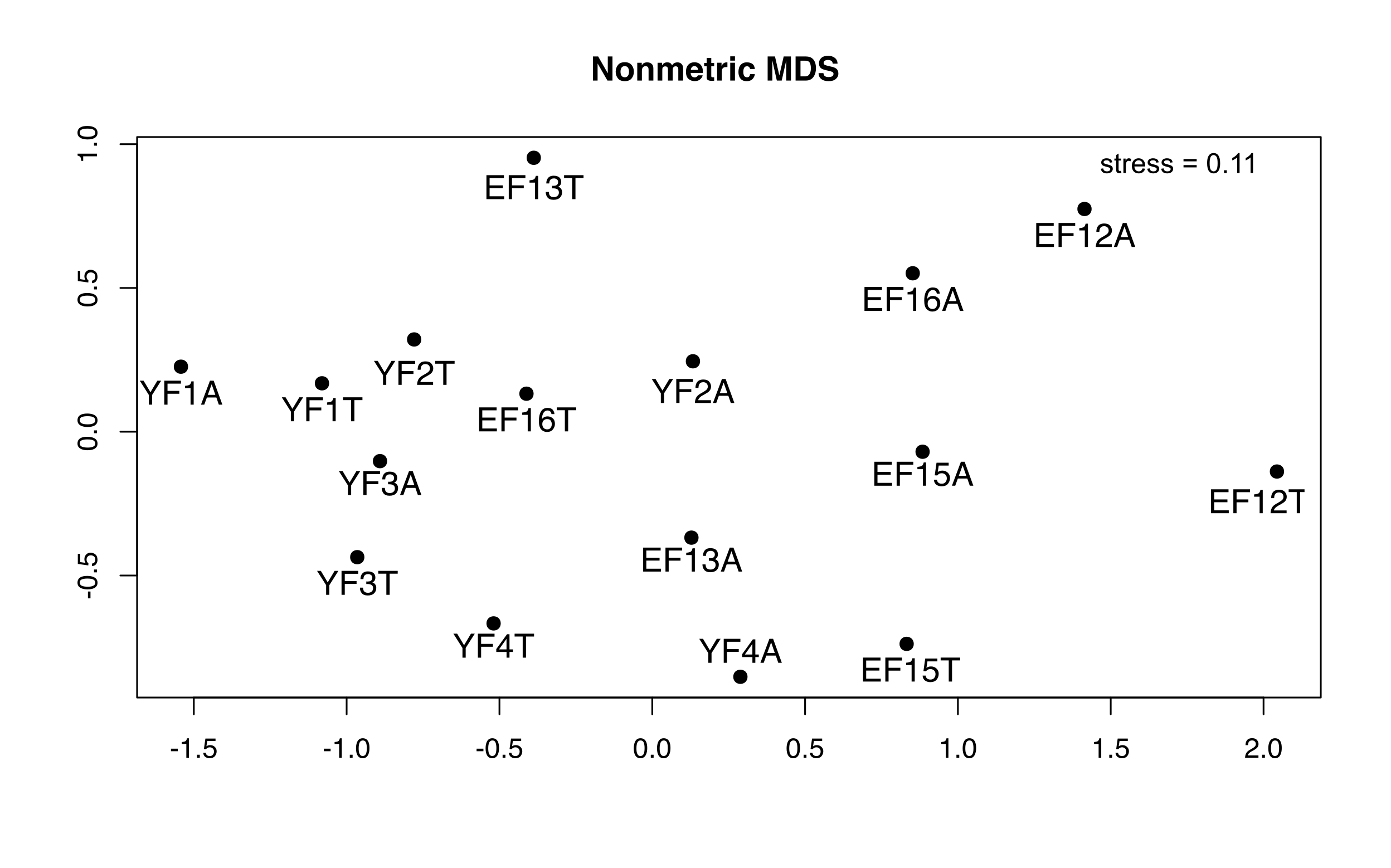


**References**

[1] R: The R Project for Statistical Computing. https://www.r-project.org/. Accessed 17 May 2018.
